# The Effect of a 10-Week Physical Activity Programme on Fundamental Movement Skills in 3–4-Year-Old Children within Early Childhood Education Centres

**DOI:** 10.3390/children8060440

**Published:** 2021-05-24

**Authors:** Ajmol Ali, Claire McLachlan, Owen Mugridge, Tara McLaughlin, Cathryn Conlon, Linda Clarke

**Affiliations:** 1 School of Sport, Exercise and Nutrition, College of Health, Massey University, Auckland 0745, New Zealand; O.Mugridge@massey.ac.nz (O.M.); C.Conlon@massey.ac.nz (C.C.); 2 Department of Sport Science and Physical Education, The Chinese University of Hong Kong, Shatin, N.T., Hong Kong, China; 3 Faculty of Education, University of Waikato, Gate 5 Hillcrest Road, Hamilton 3216, New Zealand; claire.mclachlan@waikato.ac.nz or; 4 School of Education, Federation University Australia, Ballarat, VIC 3353, Australia; 5 Institute of Education, College of Humanities & Social Sciences, Massey University, Palmerston North 4442, New Zealand; T.W.McLaughlin@massey.ac.nz (T.M.); L.Clarke1@massey.ac.nz (L.C.)

**Keywords:** pre-school, physical activity, motor skills, test of gross motor development, physical education

## Abstract

The objective of this study was to examine the effect of a 10-week physical activity (PA) programme, in early childhood education (ECE) settings, on 3 and 4-year-old children’s fundamental movement skills (FMS). A further aim was to examine FMS three-months post-intervention. The PA instructors delivered one 45 min session/week over 10 weeks, to 3- and 4-year-old children (*n* = 46), across four ECE centres. These sessions involved participation from ECE teachers. Children in the control group (CON; *n* = 20) received no PA classes and completed pre- and post-intervention assessments only. Locomotor (e.g., running/hopping) and object-control (e.g., kicking/throwing) skills were assessed using the Test for Gross Motor Development-2 (TGMD-2), before and after the intervention and, for the intervention group (EXP), at 3 months. Locomotor and object-control skills significantly improved in the EXP group, with typically no change in the CON group. The EXP group’s locomotor and object-control skills were maintained at 3 months. The 10-week PA intervention successfully improved 3- and 4-year-old children’s FMS.

## 1. Introduction

Children’s health in New Zealand is now in the bottom third of all countries [1], ranking 29th out of 30 OECD countries in 2009 [2]. New Zealand also scored 32 out of 34 countries on Unicef’s [3] ranking of children’s well-being. One third of school-age children are overweight or obese, and there are high rates and increasing prevalence of obesity in children under the age of five years [4,5]. Morbid obesity rates (BMI of ≥35 kg/m^2^) are highest in children aged 2–4 years (6%), decreasing to 3% in those aged 10–14 years [6]. Multiple factors contribute to the rates of overweight and obesity, and we live in an increasingly obesogenic society, where environmental and social factors promote increased food intake and decreased physical activity (PA), leading to higher rates of overweight and obesity [7]. Along with poor diet, a lack of PA is recognised as a major factor associated with excessive weight gain [8]. While there is an assumption that young children are naturally active, much of the PA that young children engage in is low in intensity [9]. Furthermore, young children have too many sedentary opportunities [4]. New Zealand’s PA guidelines recommend that young people (5–18 years) spend no more than two hours a day in front of a screen; however, 45% of New Zealand children (2–14 years) usually have more than two hours of screen time [10]. There is also evidence from the longitudinal Growing up in New Zealand study [11] that 2-year-olds have 1.5 h per day in sedentary screen use, raising concerns about activity levels.

Working with children to improve their gross motor skills may be one way to promote increased levels of PA. Research shows that children with better motor skills, including fundamental movement skills (FMS), which are locomotor skills such as running, hopping and jumping, and object control skills, such as throwing, catching or striking a ball, have enhanced PA levels as adolescents and adults [12]. Young children with better FMS tend to be more physically active and have greater actual competence, as well as greater self-belief or perceived competence—factors that are likely to contribute to increased participation in sport and exercise and improve later-life PA levels and health outcomes [12,13,14,15,16]. Moreover, young children’s motor skill competence and PA levels have also been associated with enhanced academic and cognitive abilities [12,17]. For example, Piek et al. [18] reported that gross motor skill ability in children aged four months to four years was a significant predictor of cognitive performance when children reached school age (6–11.5 years); however, fine motor skill was not associated. This suggests that it is important for young children to have opportunities to develop their gross motor skill competence, including FMS, in order to promote positive cognitive, academic and physical outcomes.

However, FMS are not simply developed over time but need to be coached, reinforced and practised in developmentally appropriate ways [12,19]. Therefore, the coaching, feedback and practice that occur through intervention programmes may be critical to success, as are the opportunities for young children to have access to, and participate in, physical literacy programmes. Physical literacy is the “motivation, confidence, physical competence, understanding and knowledge to maintain PA at an individually appropriate level, throughout life” [20]. Key tenets of physical literacy programmes for young children are that the experience should be personally rewarding, should emphasise the importance of maximising an individual’s potential (at an individually appropriate level), and should motivate life-long habits of being physically active [20]. Although New Zealand’s research in regard to physical literacy interventions for preschool children is limited, our study that examined the effects of a 9 week, child-centred, PA programme on the development, balance and safety skills of 12–24 month-old toddlers reported improvements in toddlers’ safety skills following the intervention [21]. Furthermore, a project that targeted New Zealand primary school children’s PA, FMS proficiency and healthy eating reported substantial improvements in their FMS, following the intervention [22]. Notably, Mitchell et al. [22] reported that, at baseline, less than half of the primary schoolchildren were proficient in the object control skills of kicking, throwing and striking, and junior children from lower-decile schools had lower FMS proficiencies than their counterparts from higher-decile schools. Implications of this are that it is important to target children’s FMS before they start school, and this is particularly relevant for those children who live in socioeconomically deprived areas.

Duncan et al. [23] implemented a “healthy homework” programme with 675 children aged 7–10 years from 16 New Zealand primary schools. Intervention schools implemented an 8-week applied homework and in-class teaching module designed to increase physical activity and improve dietary patterns. Significant intervention effects were observed for weekday physical activity at home, weekend physical activity, BMI and fruit consumption. Additional analyses revealed that the greatest improvements in physical activity occurred in children from the most socioeconomically deprived schools. The findings showed that the programme resulted in substantial and consistent increases in children’s physical activity—particularly outside of school and on weekends—with limited effects on body size and fruit consumption. Overall, the findings support the integration of compulsory home-focused strategies for improving health behaviours into primary education curricula, which has implications for ECE settings.

ECE settings have been identified as important contexts for physical literacy interventions, with potential to work with children, families and communities to help prevent obesity, by targeting practices related to children’s physical activity and nutrition [8,24,25,26]. Given that more young children are spending increasing amounts of time in childcare, appropriate PA within the ECE environment may offer many young children opportunities to develop FMS, to promote physical literacy and positive academic outcomes, to work towards equity for those children who live in low socioeconomic areas, and to combat obesity.

However, opportunities for PA may be limited for young children in ECE services in New Zealand. Although opportunities for physical play is a core requirement of licensed early childhood services [27], and “Moving confidently and challenging themselves physically” is one of the 20 learning outcomes for children in the New Zealand early childhood curriculum, Te Whāriki (p. 25), there are concerns about how consistently children have opportunities for the range of types of physical play required to achieve this learning outcome [28]. A significant barrier to appropriate PA opportunities for young children in ECE settings is teachers’ capacities to provide effective programmes. Research indicates that many teachers lack the confidence, information, knowledge and skills to provide a wide range of PA opportunities; furthermore, both pre-service and in-service professional learning and development (PLD) opportunities are limited [29,30,31]. Teachers may benefit from specific PLD opportunities that increase teachers’ PA knowledge, and that give teachers the skills and confidence they need to provide young children with appropriate PA opportunities.

The present research is part of the physical education in early childhood (PEECh) study. We have presented the findings of the effect of the intervention on teachers’ PLD elsewhere [32]. The aim of this study was to examine the effect of a 10-week PA programme in an ECE setting on 3- and 4-year-old children’s FMS abilities. A further purpose was to examine children’s FMS abilities three months after the intervention.

## 2. Materials and Methods

### 2.1. Participants

Children aged 3- and 4-years-old from four ECE centres (BestStart Educare; Auckland and Hamilton) took part in this study. Initially, 147 children were tested (at baseline) for motor skills (across the four centres). However, for several reasons (including noncompliance of children, attending fewer than four physical activity classes, nonattendance on days of testing, moving to a different area, or no longer attending day care), 66 children, 46 in the experimental (EXP) and 20 in the control (CON) groups, completed the baseline and post-intervention assessments. There was a good spread of males and females in both groups, but the participants in the CON group were significantly younger (Table 1; *p* = 0.04).

### 2.2. Study Design

Children and teachers from all four centres were involved in both EXP and CON groups. Participants in EXP also underwent data collection approximately 12 weeks following cessation of the physical activity classes (3-month follow-up).

All procedures had prior approval by the Massey University Human Ethics Committee (MUHEC Northern 15/36). Before obtaining written consent, the parents of the children who expressed an interest in the research study were fully informed about the aims, procedures and the demands that the study would place upon them, coupled with any possible risks and discomforts. Parents were reminded of their right to withdraw their child from the study at any time.

### 2.3. Physical Activity Intervention

The physical activity intervention consisted of 10 weeks of PA classes (Jumping Beans) designed to improve teachers’ knowledge related to PA, health and well-being, and to improve FMS of children. PA educators delivered one 45 min session per week over the 10-week period to 3- and 4-year-old children in the EXP group. The curriculum was designed to be fun, engaging and educational, for both children and teachers. The classes were limited to a maximum of 25 children to allow for engagement with teachers and PA instructors.

The 10-week programme was based on strengthening locomotor skills using fundamental movement patterns (locomotion, statics, rotation, springing, landing, manipulative and swing). To encourage manipulative skills, a unique ball skill was also associated with each locomotor skill. The ball skills used were an underhand roll, an overhand throw, catching, kicking, striking a stationary ball and dribbling a ball. The first 6 weeks of the intervention were centred around one locomotor skill and one ball skill, which were taught in a 45 min session. The sessions in the remaining 4 weeks were designed to reinforce difficult skills from the previous 6 weeks.

Each 45 min session was based on an animal (e.g., ‘gallop like a horse’) to appeal to the children. The 45 min sessions were split into 3 blocks: 5 min for mat time, 35 min for equipment (7 × 5 min blocks) with each equipment piece relating to one of the seven fundamental skills, and 5 min for cool down. Mat time was used to introduce the programme for the week and demonstrate the locomotor skill to the children. A colouring-in picture, programme notes and a take-home activity were also given to the children based on the relevant animal. Thereafter, the equipment was used to strengthen skills useful for the locomotor skill that week. The cool-down period was used for music and movement, bubbles and ‘fun-chute’ activities to animal-themed music.

Children and teachers within the CON group received only guidance for PA. For ethical reasons, the children in the CON group were provided with the 45 min session per week over a 10-week period, after the intervention was completed in the EXP group.

### 2.4. Test of Gross Motor Development, 2nd Edition (TGMD-2)

The (TGMD-2) [31] was used to assess FMS before and after the intervention. The TGMD-2 is suitable for 3–10-year-olds and uses skill-specific performance criteria to assess locomotor skills, such as running and hopping, and object control skills, such as kicking and throwing [31].

An instructor demonstrated each TGMD-2 task (using specific criteria) on two occasions before the child made two attempts at each task. Four instructors were used over the 28 TGMD-2 assessments days. Each instructor underwent training moderated by a primary assessor using physical demonstration, verbal instruction and the TGMD-2 examiner’s manual and visual diagrams.

The raw scores for locomotor and object control were then tallied by the instructors and the Standard Score, Percentile and Age Equivalent scores calculated using the TGMD-2 conversion tables [33]. The primary assessor, who was also responsible for scoring each child’s TGMD-2 assessment on all 28 testing days, checked the scoring.

Raw data that have been converted, based on specific age of the child, are termed ‘standard scores’. These scores allow comparison between locomotor and object control subtests. The percentiles (or percentile ranks) indicate the percentage of the distribution equal to or below a particular score. For example, a percentile score of 60 means that 60% of the standardised sample scored at or below the examinee’s score. It should be noted that these values are based on standardised percentile data from the USA. The age equivalents for tests of developmental abilities have been termed ‘developmental ages’ [33]. The TGMD-2 age equivalents’ data provide an estimation of how the subtest scores relate to typical age.

Pilot testing was carried out prior to visiting the centres to ensure the primary assessor recognised the successful completion or failure of each skill criteria. Periodically throughout the testing period, an additional instructor also scored the child’s TGMD-2 assessment to check the reliability and reproducibility of the primary assessor’s scoring.

Each testing environment within the four centres was assessed for health and safety risks before each TGMD-2 session took place. Each room was chosen to match the requirements of each locomotor and object control skill whilst reducing the noise level, interruptions and distractions that could occur during a child’s assessment.

### 2.5. Statistical Analysis

Statistical analysis was conducted using IBM SPSS version 21. Data were examined using mixed-method repeated-measures analysis of variance (ANOVA), for within-subjects (baseline vs. post-intervention) and between-subject (intervention vs. control) effects. Mauchly’s test of sphericity was used to determine whether the assumption of sphericity was being violated by the data. Where this did occur, the Huynh–Feldt correction was applied. One-way ANOVA was used to examine the time of assessment effects (baseline vs. post-intervention vs. 3-month follow-up) in EXP only. When differences were found by ANOVA, paired Student’s t-tests, using the Holm–Bonferroni adjustment, were used to ascertain where the differences lay. The Student’s *t*-test for independent data was also used to examine differences between trials. Effect size (Cohen’s d) was used to report practical significance; a large effect size was determined as 0.8, medium as 0.5 and small as 0.2 [32]. Relationships between independent variables were assessed using Pearson’s correlation. A small (weak) correlation was defined as ±0.10 to ±0.29, medium (moderate) correlation as ±0.30 to ±0.49 and large (strong) as ±0.50 to ±1.00 [32]. Data are presented as means ± standard deviation (SD). The level of significance was accepted at *p* < 0.05.

## 3. Results

### 3.1. TGMD-2 Standard Scores

**Figure 1:**
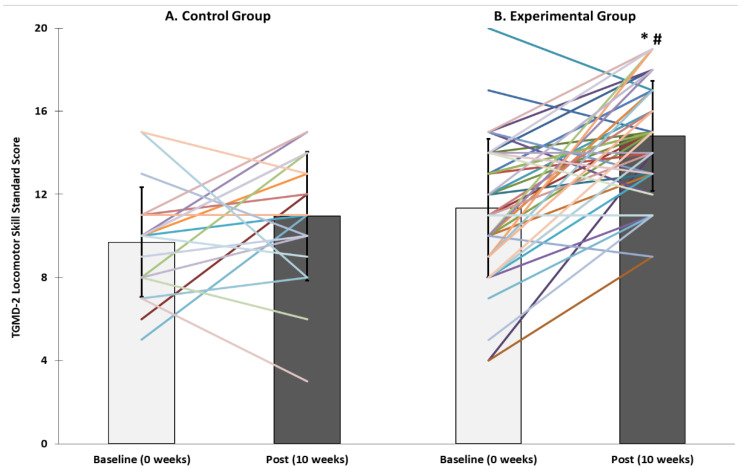
Test of Gross Motor Development (TGMD-2) locomotor skill standard scores pre-and post-intervention in (**A**) Control (*n* = 20) and (**B**) Experimental (*n* = 46) groups. * Significantly higher than baseline (*p* < 0.05). ^#^ significantly higher than control group (*p* < 0.05).

There was a significant time*treatment effect for locomotor standard scores (*p* < 0.001). There was no difference in baseline locomotor standard scores between CON and EXP groups, indicating that all participants were at a similar level at the start of the study. There was no difference in locomotor standard scores from pre- to post-intervention in CON (*p* = 0.15). However, locomotor standard scores improved from baseline to post-intervention in EXP (*p* < 0.001) and were higher post-intervention in EXP than CON (*p* < 0.01; Figure 1).

**Figure 2:**
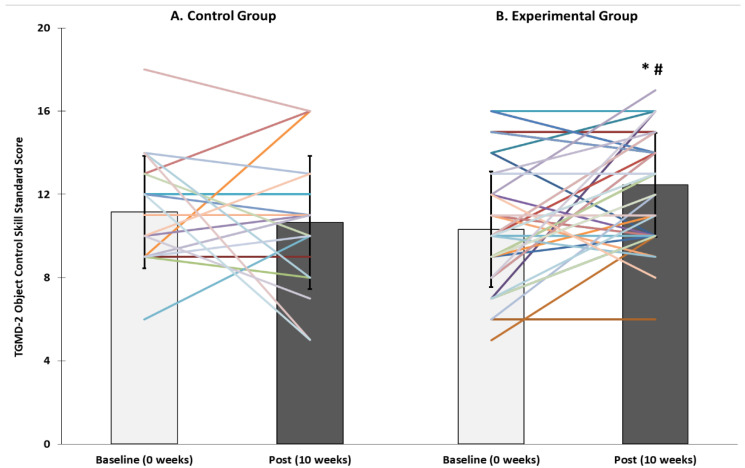
Test of Gross Motor Development (TGMD-2) object control skill standard scores pre- and post-intervention in (**A**) Control (*n* = 20) and (**B**) Experimental (*n* = 46) groups. * Significantly higher than baseline (*p* < 0.05). ^#^ significantly higher than control group (*p* < 0.05).

There was a significant time*treatment effect for object control standard scores (*p* < 0.001). There was no difference in baseline object control scores between CON and EXP trials (*p* = 0.27) or between baseline and post-intervention in CON (*p* = 0.56). However, object motor standard scores improved from baseline to post-intervention in EXP (*p* < 0.001) and were higher post-intervention in EXP than CON (*p* = 0.03; Figure 2).

### 3.2. TGMD-2 Percentile Scores

There was no interaction effect for locomotor percentile scores. Nevertheless, there was a main effect of time (*p* < 0.001), indicating that all children improved their abilities. Furthermore, there was a main effect of treatment (*p* < 0.001), with children in the EXP trial in the higher percentiles (Table 2). As opposed to locomotor percentile scores, there was a significant interaction effect of treatment*time for object control percentile scores (*p* < 0.001). Post-hoc analysis showed that there was no difference between baseline and post-intervention in CON (*p* = 0.63). However, percentile ranks improved in EXP (*p* < 0.001), indicating that physical activity classes improved object control abilities by 26.1 (±28.0) percentile ranks (Table 2). Furthermore, there was a significant difference between CON and EXP post-intervention (*p* =0.02), with children in EXP classes improving object control skills by ~20 percentile ranks.

### 3.3. TGMD-2 Age-Equivalent Scores

There was a significant interaction effect for locomotor age equivalents (*p* = 0.02). There was no difference in locomotor age-equivalent scores between baseline and post-intervention in CON (*p* = 0.08). However, the mean age equivalent increased from 5.1 (±1.8) to 7.1 (±2.0) years in the EXP trial (*p* < 0.01). Furthermore, at the end of the intervention, the age equivalent for EXP (7.1 ± 2.0 years) was significantly higher than that for CON (4.8 ± 1.6 years; *p* < 0.01). There was no difference in object control age equivalents between baseline and post-intervention scores for CON (*p* = 0.91). However, the mean age equivalent for object control skills improved from 4.4 (±1.5) to 5.6 (±1.5) years in EXP (*p* < 0.001). Furthermore, the post-intervention age equivalents were significantly higher for EXP (*p* = 0.02), indicating that the PA classes improved the age equivalent for object control skills by approximately one year.

### 3.4. TGMD-2 Follow-Up Scores

The 3-month follow-up scores in EXP were higher than baseline (*p* < 0.05). There was no difference between post-intervention and 3-month follow-up scores for locomotor (*p* = 0.62) or object control (*p* = 0.34) skills, indicating that children maintained their skills when the physical activity instructors were not present. Similar results were also observed for locomotor percentile, object control percentile, as well as locomotor age-equivalent and object control age-equivalent scores (Table 3).

## 4. Discussion

The aim of this study was to examine the effect of a 10-week PA intervention on motor skills in children within ECE. The physical activity programme improved fundamental movement skills in 3- and 4-year-old children, relative to the control group, in both locomotor ability (including running, jumping and hopping) and object control skills (including ball-handling skills of throwing, catching and bouncing). Children who undertook PA classes increased their locomotor abilities by an average of 20 percentile rankings and two age-equivalent years, and object control skills by 20 percentile rankings and one age-equivalent year. A secondary aim was to examine children’s FMS ability three months after the intervention. Locomotor and object control abilities were maintained by the children in the EXP group during the follow-up period.

We report data in terms of TGMD-2 standard scores, percentile scores and age-equivalent scores. Overall, children’s locomotor and object control skills improved because of the intervention. These results are consistent with a growing body of research that has reported improvements in young children’s FMS following PA or FMS intervention. For example, Hardy et al. [24] reported that children’s (mean age 4.4 years) FMS scores significantly improved in the intervention group (compared with control group) following the implementation of a professional development program designed to support ECE teachers to promote young children’s healthy eating and physical activity. Bardid et al. [33], who implemented a motor skill programme provided by trained instructors in childcare settings (3–8-year-olds), reported a significant gain in locomotor and object control skills compared to the control group. It can be difficult to directly compare results of interventions because of differences such as intervention type, methodology and assessment tools. However, a meta-analysis [19] reported significant and similar improvements in young children’s object control (d = 0.41; *n* = 12; *p* < 0.001) and locomotor skills (d = 0.45, *n* = 9; *p* < 0.001) following PA or FMS-type interventions.

On average, children in the present study completed seven 45 min *Jumping Beans* sessions during the 10-week intervention (children taking part in three or fewer classes were excluded from the data analysis). It is notable that significant FMS improvements were achieved in the short intervention time of 10 weeks. In comparison, over a 30 week time frame, the Bardid et al. [33] intervention consisted of a single PA session (~60 min per week), and Jones et al. [34] implemented Jump Start sessions (20 min), in addition to unstructured PA sessions, three times per week. Interestingly, the meta-analysis of Logan et al. [19] did not find an association between the effect size of intervention improvements and the duration of the intervention (in minutes), suggesting that a longer intervention does not necessarily mean stronger outcomes. That said, there are many other variables, including the frequency and type of intervention, that are likely to impact on success. There is limited information about the impact of PA intervention dose, including relationships between the length of an intervention and successful outcomes for children, and longer-term, or residual, effects.

A key purpose of the present study was to investigate any residual effects of the PA programme. Follow-up assessments showed that locomotor and object control abilities were maintained by the EXP children (*n* = 25) three months after Jumping Beans classes finished. Of the few studies that have investigated residual effects of FMS interventions for young children, Jones et al. [34] assessed children’s (3–5 years, *n* = 60) gross motor skills six months after an intervention, and they reported small to medium treatment effects for gross motor skill outcomes, with all (except for the catch) showing positive trends in favour of the intervention group. Reilly et al. [35] reported significant improvements in gross motor skills (measured with the Movement Assessment Battery for Children) six months after a PA intervention.

A further consideration, and variable among comparative studies, is whether interventions are teacher-led or directed by a trained instructor. For example, Bardid et al. [33] used a trained instructor, while Jones et al. [34] trained teachers to lead the PA/FMS programmes. In the present study, *Jumping Beans* PA educators facilitated the intervention sessions; however, this was combined with teachers’ involvement during each session with a focus on teachers learning new skills, plus three professional development workshops on physical literacy, physical activity and nutrition [30]. It is possible that this dual approach contributed to the positive outcomes. The advantages of teacher-led programmes are that they are likely to be more economically and practically sustainable, and teachers can, potentially, offer children who are enrolled in early education and care centres ongoing FMS coaching. Whether facilitated by a teacher or an outside coach, a common feature of successful interventions may be the planned and intentional nature of the FMS programme. Evidence continues to show that FMS are not simply developed over time but need to be coached, reinforced and practiced in developmentally appropriate ways [12,19]. Therefore, the coaching, feedback and practice that occur through intervention programmes may be critical to success, as are the opportunities for young children to participate in appropriate physical literacy programmes.

Given the potential for PA interventions to improve young children’s FMS, and given the links between FMS competence and later-life PA [15], interventions may be key to addressing issues related to sedentary behaviour and obesity. Furthermore, ECE services have potential as optimal contexts in which to deliver physical activity interventions to many young children. However, as Wolfenden et al. [8] highlight, the proper implementation of policies and practices, and the promotion of ECE teachers’ physical literacy knowledge, skills and attitudes, are essential if ECE services are to succeed in any endeavour to promote young children’s physical literacy and/or obesity prevention. While evidence continues to report that PA/FMS interventions can result in positive outcomes for children, we need to know how to successfully implement physical literacy interventions in ECE settings, in order to promote positive outcomes for children. Thus, this study provides evidence and insight into how issues related to preschool children’s FMS and PA might be addressed in the New Zealand context. Notably, the context of ECE settings as venues for teacher-led interventions, and the significant improvements in young children’s FMS, which can be attributed to the Jumping Beans’ programme, provide positive future directions for physical literacy programmes, practice and policies. There are also implications for further research, especially regarding how successful physical literacy programmes might be implemented as part of a quality ECE environment. Along these lines, although in a primary school context, another New Zealand study [22], which focused on primary-school-aged children (*n* = 598 at follow-up), reported that tailored FMS programmes, as part of a multi-component, multi-school intervention, improved children’s FMS competencies. Interestingly, Mitchell et al. [22] reported that young children from low-decile schools were less proficient in FMS than their higher-decile counterparts. One of the reasons for the socioeconomic disparity may be that obesity levels are higher in poorer socioeconomic areas [5,6]; however, following the intervention, the FMS of children from the lower-decile schools improved to the extent that the gap was substantially reduced [22]. This suggests that it may be particularly important to introduce physical literacy programmes into ECE services in low socioeconomic areas, to address disparities before children begin school. In the current study, we worked with 3 and 4-year-old children; however, younger children, and the teachers that work with them, may benefit from physical literacy programmes to a greater extent, as the very early years represent a time span of more rapid development.

There are limitations of this study which should be considered. At baseline and post-intervention, 46 children were available and eligible for assessment but only 25 children were available for assessment at the 3-month follow-up. One of the reasons for the high dropout rate was transience—many of the participating children had left the early education services. While this dropout rate has resulted in a low sample size, it does highlight transience as a potential issue to address when implementing physical literacy programmes in early childhood settings, particularly in low socioeconomic areas where there is likely to be higher rates of transience [36]. In addition, while FMS are important, another aspect of a physical literacy intervention is the impact it may have on children’s PA levels.

In future research, a larger baseline sample size may be needed to offset possible drop-out rates. While only children’s motor skill and PA data are reported here, a key aspect of this study was the involvement of the ECE teachers, who underwent professional development as part of this intervention. Further exploration into the important roles of ECE teachers to promote children’s FMS and physical activity is a valuable direction for future research. Moreover, unstructured play can promote healthy child development and promote learning [37]. It was beyond the scope of this study to explore these aspects further, but future studies should further examine the effect of structured PA (such as the 10 week intervention presented here) on the type and amount of play in children within ECE. Furthermore, while there has been research showing that improved motor skills enhance various aspects of physical activity, academic abilities and health outcomes, no studies have examined all these aspects together, and there is no New Zealand-specific research in this area. Providing firm data linking physical literacy, physical activity and improved academic and health outcomes in the early childhood space is vital to increase awareness of this topic within ministries of sport, health and education.

## 5. Conclusions

The 10-week physical activity intervention within early childhood centres significantly improved 3- and 4-year-old children’s locomotor and object control skills. Our findings show that even a short intervention can improve young children’s FMS, although we need to know more about how the frequency, length and facilitation of interventions, along with teachers’ PLD, might affect various outcomes for children. Furthermore, children’s skills were maintained in a 3-month follow-up assessment (when no specific physical activity sessions were provided). The physical activity instructors worked with teachers to increase the teacher’s knowledge and confidence and to enhance teaching skills related to supporting and coaching young children’s FMS. Together, these appear to be important aspects of a successful physical literacy intervention. 

## Figures and Tables

**Table 1:** Demographic information of participants.

Variable	Experimental Group (*n* = 46)	Control Group (*n* = 20)
Age at baseline (years; mean ± SD)	4.1 ± 0.6	3.8 ± 0.5 ^1^
Sex		
Male (*n*)	24	13
Female (*n*)	22	8
Number of PA classes attended (*n*; mean ± SD)	7.0 ± 1.7	None

SD: Standard deviation. ^1^ Significantly younger than experimental group (*p* < 0.05).

**Table 2:** Test of Gross Motor Development (TGMD-2) locomotor and object control scores in experimental (*n* = 46) and control (*n* = 20) groups.

Variable	Experimental Group (*n* = 46)			Control Group (*n* = 20)		
	Standard Score	Age-Equivalent	Percentile Ranks	Standard Score	Age-Equivalent	Percentile Ranks
Locomotor skill						
Baseline	11.3 (3.3)	5.1 (1.8)	62.2 (29.9)	9.7 (2.6)	4.1 (1.4)	46.1 (26.6)
Post	14.8 (2.6) ^1,2^	7.1 (2.0) ^1,2^	88.3 (15.3)	11.0 (3.1)	4.8 (1.6)	60.9 (29.3)
Mean change	3.5 (3.4) ^2^	2.0 (2.1) ^2^	26.1 (28.0)	1.3 (3.6)	0.8 (1.9)	14.8 (35.0)
Object control skill						
Baseline	10.3 (2.8)	4.4 (1.5)	52.1 (28.1)	11.2 (2.7)	4.7 (1.6)	60.0 (26.0)
Post	12.5 (2.5) ^1,2^	5.6 (1.5) ^1,2^	73.6 (22.9) ^1,2^	10.7 (3.2)	4.7 (1.4)	55.8 (29.6)
Mean change	2.1 (3.0) ^2^	1.2 (1.6) ^2^	21.5 (31.1) ^2^	−0.5 (3.8)	−0.1 (1.9)	−4.2 (37.9)

^1^ Significantly higher than baseline (*p* < 0.05). ^2^ significantly higher than control group (*p* < 0.05).

**Table 3:** Test of Gross Motor Development (TGMD-2) locomotor and object control scores in experimental group of children who took part in all three sessions (*n* = 25).

Variable	Experimental Group (*n* = 25)		
	Standard Score	Age-Equivalent	Percentile Ranks
Locomotor skill			
Baseline	11.5 (3.3)	4.9 (1.8)	63.2 (29.6)
Post	14.7 (1.8) ^1^	6.8 (1.5) ^1^	91.2 (8.5) ^1^
3-month follow-up	15.7 (2.6) ^1^	7.3 (2.2) ^1^	92.5 (8.3) ^1^
Mean change ^2^	0.9 (2.9)	0.3 (2.0)	1.4 (10.1)
Object control skill			
Baseline	10.6 (2.8)	4.5 (1.7)	54.6 (28.2)
Post	12.6 (2.3) ^1^	5.4 (1.4) ^1^	75.5 (22.2) ^1^
3-month follow-up	13.8 (2.1) ^1^	5.8 (1.6) ^1^	84.5 (16.4) ^1^
Mean change ^2^	1.3 (2.6)	0.3 (1.3)	10.7 (22.0)

^1^ Significantly higher than baseline (*p* < 0.05). ^2^ Mean change (3-month vs. Post).

## Data Availability

All relevant data are presented in this manuscript.
